# Case Report: COVID-19 Masquerading as an Acute Surgical Abdomen

**DOI:** 10.4269/ajtmh.20-0559

**Published:** 2020-06-09

**Authors:** Ashraf O. E. Ahmed, Mohamed Badawi, Khalid Ahmed, Mouhand F. H. Mohamed

**Affiliations:** 1 Internal Medicine Department, Hamad Medical Corporation, Doha, Qatar;; 2 Infectious Disease Department, Hamad Medical Corporation, Doha, Qatar;; 3 Acute Care Surgery Department, Hamad Medical Corporation, Doha, Qatar

## Abstract

SARS-CoV-2 infection can present with various clinical features, among which gastrointestinal manifestations such as nausea, diarrhea, vomiting, and mild abdominal pain have been reported. Recognition of rare presentations of SARS-CoV-2 infection has increased over time. These atypical and rare presentations may lead to difficulties in establishing the diagnosis in a timely manner; furthermore, they may lead to unnecessary investigations, extended hospital stays, adverse outcomes, and more strain on healthcare resources. We present three cases admitted to our hospital with a picture that mimicked an acute abdomen, necessitating surgical assessment and evaluation. All cases turned out to be SARS-CoV-2 positive and did not require surgical management. We discuss the management course, highlight the importance of abdominal symptoms in the setting of COVID-19, and discuss the implications of this association for medical practice amid the current pandemic in both resource-rich and resource-limited settings.

## INTRODUCTION

COVID-19 typically not only affects the respiratory system but can also affect other body organs. The manifestations differ according to the system predominantly affected. A meta-analysis including data of around 2,000 patients revealed that fever (89%), cough (69%), and muscle aches with fatigue (36%) are the most prevalent symptoms, whereas nausea, vomiting, and diarrhea were less common (4–5%).^1^ This analysis did not specify the prevalence of abdominal pain, but that can be ascertained from another review exploring the incidence of gastrointestinal (GI) manifestations, depicting a 2–6% prevalence of abdominal pain among patients with COVID-19.^2^ Nonetheless, acute abdominal pain is uncommon. We share our experience with three cases presenting with acute abdominal pain necessitating, abdominal imaging, and surgical evaluation.

## CASE DESCRIPTIONS

**Figure 1.**
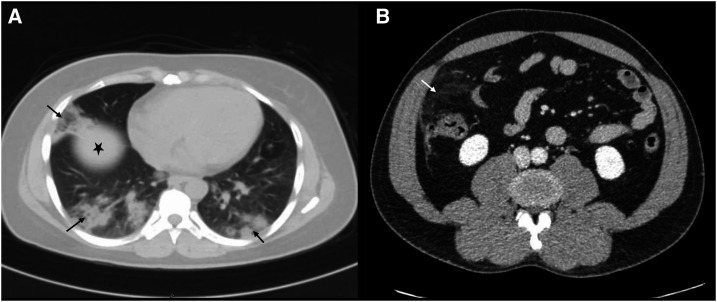
(**A**) Abdominal computed tomography (CT) scan of case 1 showing lower lung zones with patchy bilateral areas of consolidation and ground-glass attenuation (black arrows). Liver (star). (**B**) CT scan of the abdomen of case 2 showing diffuse stranding of the right hypochondrial omentum/mesentery surrounding a focal fat dense ovoid area (white arrow) suggestive of an underlying epiploic appendagitis.

The first case was a 40-year-old woman who presented with a 2-day history of a vague right lower quadrant (RLQ) abdominal pain that was severe on the day of admission. On further inquiry, she reported fever, nausea, and vomiting but no diarrhea. Examination revealed RLQ abdominal tenderness, however, with no guarding or rigidity. Laboratory workup was unremarkable, except for a mild elevation of the C-reactive protein (CRP) (Table 1). Computed tomography (CT) scan of the abdomen showed no evidence of appendicitis but bilateral basal lung consolidations (Figure 1). Thus, COVID-19 suspicion arose and was confirmed subsequently with nasopharyngeal swab testing positive for SARS-CoV-2 using real-time PCR (RT-PCR). Consequent to this, a surgical evaluation was not sought further. The patient was treated as COVID-19 pneumonia (hydroxychloroquine, darunavir/cobicistat, and azithromycin). The abdominal symptoms improved on day 10 of the admission. She remained in good health after completing her treatment course until she was discharged 31 days after the first positive COVID-19 PCR. The COVID-19 local management protocol at the time mandated the negativity of two consecutive COVID-19 PCR tests before discharging the patients; this caused the prolonged hospital stay.

**Table 1:** Summary of clinical, laboratory, imaging characteristics, and outcomes of three cases with acute abdominal pain and COVID-19 infection

Patient	Respiratory symptoms, oxygen saturation	Abdominal pain region	WBC (×10^3^/uL)	CRP (mg/L)	Ferritin (ug/L)	Lipase (U/L)	Computed tomography abdomen	Chest X-ray	COVID-19 PCR	Duration to recovery (days)
Forty-year-old female (patient 1)	None, 99% on ambient air	RIF	3	14.4	291	Not done	Upper cut: Bilateral basal lung consolidation	Coarse broncho-vascular markings	Positive	31
Forty-four –year-old male (patient 2)	Dry cough, 98% on ambient air	Right upper quadrant	9.6	35	283.0	Not done	Epiploic appendagitis	Small pneumonic patch in the left lower lobe	Positive	38
Fifty-three–year-old male (patient 3)	None, 98% on ambient air	Epigastric	6.5	82	Not done	230	Normal	Bilateral basilar infiltrates	Positive	29

Reference values: WBC = white blood cells (4–10 × 10^3^/uL); CRP = C-reactive protein (0–5 mg/L); ferritin = 30–490 ug/L; lipase = 8–78 U/L.

The second case was a 44-year-old man who presented with excruciating right upper quadrant (RUQ) abdominal pain. Further questioning revealed only a 5-day history of mild dry cough preceding the pain. No other symptoms were present. The abdominal examination revealed guarding and right hypochondriac tenderness but no organomegaly. Other systemic examinations, including the respiratory system, were unremarkable. Initial laboratory tests were significant for a mild CRP rise (Table 1). Abdominal ultrasound ruled out cholecystitis. An abdominal CT scan was performed for further evaluation and showed features suggestive of right hypochondria epiploic appendagitis versus omental infarction, with clear lung basal segments (Figure 1). Surgical evaluation ceased at this point, and COVID-19 evaluation (local hospital protocol for admitted patients) commenced, and, subsequently, the infection was confirmed (RT-PCR). The patient was treated with a short course of azithromycin and hydroxychloroquine. His symptoms resolved in a few days; he remained asymptomatic and was discharged after 38 days (two negative RT-PCR tests).

The third case was a 53-year-old man known to have diabetes, hypertension, and end-stage renal disease. The chief complaint was severe mid-upper abdominal pain, associated with nausea, vomiting, and diarrhea of 2-day duration. A systemic query was insignificant otherwise. The examination was significant for fever, and mild epigastric tenderness that did not correlate with the severe pain the patient described. Other systemic examinations, including the respiratory system, were unremarkable. Laboratory evaluation revealed a mild CRP rise and an elevated lipase 230 UL (normal range 8–78 U/L). He was labeled as a case of acute pancreatitis, and conservative management commenced. An abdominal CT was performed two days later because of non-improvement and did not show evidence of pancreatitis. The case was discussed with infectious disease colleagues, and, in light of our experience with the previous two cases, we requested a chest X-ray, which showed patchy infiltrates in the left upper and lower lung zones. Hence, the patient was tested for COVID-19 RT-PCR, and it was reported to be positive. The patient was started on COVID-19 pneumonia treatment (lopinavir/ritonavir, hydroxychloroquine, and azithromycin). The pain resolved within the first week of the admission. Thereafter, he remained asymptomatic until hospital discharge on day 29.

Anti–COVID-19 therapeutic agents varied between cases. This was according to local guidance at the time of each hospitalization and to medication availability. All three cases did not need critical care unit admission, and their hospitalization course was uncomplicated otherwise.

## DISCUSSION

The acute abdomen is a surgeon’s challenge. It can lead to grave consequences if not identified early and treated promptly.^3^ Vague presentations of COVID-19 in the midst of this pandemic, as depicted by our cases, make this evaluation even more challenging. We shared our experience with three cases that constituted an initial diagnostic dilemma. History of fever in cases 2 and 3 and the history of mild cough in case 2 was useful in reaching the diagnosis. However, the dilemma persisted until the management was commenced, and patients’ pain settled.

Saeed et al.^4^ performed a retrospective analysis of all acute abdominal pain cases admitted to their institute. Their evaluation revealed that nine of 79 cases tested positive for COVID-19. These patients had no respiratory symptoms. Six of nine cases had a chest CT scan abnormality. The authors postulated the role of angiotensin-converting enzyme-2 (ACE2) receptor in the pathogenesis of abdominal pain. The virus binds to the ACE2 receptor, and the receptor can be found in the lungs and various GI system structures, including the intestines.^5^ Bhayana et al.^6^ reported abdominal imaging findings of COVID-19 patients. In their report, four patients were found to have findings suggestive of nonocclusive mesenteric ischemia. Moreover, on laparotomy, an atypical yellow color was present. Bhayana et al. suggested a role of ACE2 receptor, and the possibility of direct vasculature invasion by the virus or occlusion resulting from the microthrombus formation.

Consequent to this, microthrombus formation leading to pain can also be added to the causes of pain in our patients. It may have led to appendagitis in case 2 and mild undiagnosed mesenteric ischemia in case 3 (hinted by severe abdominal pain and disproportionately mild abdominal tenderness as elicited by physical examination). Pain in cases 1 and 3 could be radiating pain from lower lung involvement. However, the pain in case 1 was only limited to the RLQ, making it less likely to be the sole cause of this patient’s pain. Pancreatitis has been reported in association with COVID-19.^7^ This may have been the cause of pain in case 3; however, he had mild lipase rise, no significant abdominal tenderness, and CT scans showing no evidence of pancreatitis.

Based on this limited experience, it seems prudent to have a low threshold for COVID-19 infection when dealing with acute abdominal pain, especially atypical. This is even of more importance in resource-limited settings, where there is a need to preserve valuable resources and avoiding burdening the patients and health systems. Notwithstanding this, appropriate evaluation of acute abdominal cases should proceed, maintaining personal protective equipment, as not to miss relevant surgical cases. Abdominal CT scan with the involvement of mid to lower cuts of the chest is of additional value in the evaluation of such cases whenever available. As time passes, more data will accrue that will help us better understand this disease and its implications on various medical and surgical services.

In conclusion, severe abdominal pain can be the presenting feature of COVID-19. Clinicians should be aware of this presentation and should have a low threshold to diagnose it amid the current pandemic. Overlooking such diagnosis may lead to improper triage, leading to in-hospital SARS-CoV-2 transmission. In addition, it may lead to improper investigation and management that may be invasive.
